# A bioinformatics approach to distinguish plant parasite and host transcriptomes in interface tissue by classifying RNA-Seq reads

**DOI:** 10.1186/s13007-015-0066-6

**Published:** 2015-05-03

**Authors:** Daisuke Ikeue, Christian Schudoma, Wenna Zhang, Yoshiyuki Ogata, Tomoaki Sakamoto, Tetsuya Kurata, Takeshi Furuhashi, Friedrich Kragler, Koh Aoki

**Affiliations:** Graduate School of Life and Environmental Sciences, Osaka Prefecture University, 1–1 Gakuen–Cho, Naka–Ku, Sakai, Osaka, 599–8531 Japan; Max Planck Institute of Molecular Plant Physiology, Wissenschaftspark Potsdam-Golm, Am Mühlenberg 1, Potsdam, 14476 Germany; Plant Global Education Project, Graduate School of Biological Sciences, Nara Institute of Science and Technology (NAIST), 8916–5 Takayama, Ikoma, 630–0192 Japan; Metabolic Systems Research Team, RIKEN Center for Sustainable Resource Science (CSRC), 1–7–22 Suehiro, Tsurumi, Yokohama, 230–0045 Japan; Present Address: Bioinformatics Group, The Sainsbury Laboratory, Norwich Research Park, Norwich, NR4 7UH UK; Present Address: Department of Molecular Systems Biology (Ecogenomics and Systems Biology), Vienna University, Althanstraße 14, Vienna, A–1090 Austria

**Keywords:** *Cuscuta japonica*, *Cuscuta reflexa*, Classification, Parasitic plant, Transcriptome, Parasite-host interaction

## Abstract

**Background:**

The genus *Cuscuta* is a group of parasitic plants that are distributed world-wide. The process of parasitization starts with a *Cuscuta* plant coiling around the host stem. The parasite’s haustorial organs then establish a vascular connection allowing for access to the phloem content. The host and the parasite form new cellular connections, suggesting coordination of developmental and biochemical processes. Simultaneous monitoring of gene expression in the parasite’s and host’s tissues may shed light on the complex events occurring between the parasitic and host cells and may help to overcome experimental limitations (i.e. how to separate host tissue from *Cuscuta* tissue at the haustorial connection). A novel approach is to use bioinformatic analysis to classify sequencing reads as either belonging to the host or to the parasite and to characterize the expression patterns. Owing to the lack of a comprehensive genomic dataset from *Cuscuta* spp., such a classification has not been performed previously.

**Results:**

We first classified RNA-Seq reads from an interface region between the non-model parasitic plant *Cuscuta japonica* and the non-model host plant *Impatiens balsamina*. Without established reference sequences, we classified reads as originating from either of the plants by stepwise similarity search against *de novo* assembled transcript sets of *C. japonica* and *I. balsamina*, unigene sets of the same genus, and cDNA sequences of the same family. We then assembled *de novo* transcriptomes from the classified read sets. We assessed the quality of the classification by mapping reads to contigs of both plants, achieving a misclassification rate low enough (0.22-0.39%) to be used reliably for differential gene expression analysis. Finally, we applied our read classification method to RNA-Seq data from the interface between the non-model parasitic plant *C. japonica* and the model host plant *Glycine max*. Analysis of gene expression profiles at 5 parasitizing stages revealed differentially expressed genes from both *C. japonica* and *G. max*, and uncovered the coordination of cellular processes between the two plants.

**Conclusions:**

We demonstrated that reliable identification of differentially expressed transcripts in undissected interface region of the parasite-host association is feasible and informative with respect to differential-expression patterns.

**Electronic supplementary material:**

The online version of this article (doi:10.1186/s13007-015-0066-6) contains supplementary material, which is available to authorized users.

## Background

In angiosperms, approximately 4000 species are parasitic to some extent [1]. Parasitic plants have evolved from at least 11 independent clades [2]. They depend, partly or entirely, on a host plant for acquisition of water and nutrients. The ability to consume water and nutrients from the host plant affects the appearance and metabolism of parasitic plants. In general, parasitic plants either partially lost the capacity of photosynthetic production (hemiparasitic plants) or entirely depend on host plants (holoparasitic plants) [3].

The genus *Cuscuta* is a prominent group of parasitic plants. It consists of 150–200 species that are distributed world-wide [4]. Some *Cuscuta* spp. are known to infest fields, thereby leading to crop losses. Although seedlings of *Cuscuta* are self-sufficient, mature plants have no roots, and their leaves are reduced to small scales. Parasitism of *Cuscuta* starts with sensing the host plant and coiling around the host stem. This action is followed by formation of prehaustorium structures from meristematic cells [5]. Invasion of the host tissue by the haustorium is initiated by production of a set of enzymes degrading the host cell wall [6] and inducing a host defense response (also reported for herbivores and pathogens [7]). According to the degree of defense response of the host plant to prevent the haustorium from reaching the vascular tissue or from establishing a functional conduit, the interaction between parasite and host plants can be classified as compatible or incompatible [8,9]. In a compatible host, a *Cuscuta*-host feeding connection is usually established by the formation of new vascular tissue connecting the pre-existing host vasculature to the *Cuscuta* vasculature. Dye tracer experiments showed both an apoplastic [8] and a symplastic exchange [10] of small molecules between the species. Additionally, the transfer of macromolecules such as mRNA [11,12], and siRNA [13] as well as viruses [14] indicates the existence of a symplastic parasite-host interface. Furthermore, microscopy studies demonstrated the presence of protoxylem cells in the interface between *Cuscuta* and host tissues [8]. Even when *Cuscuta* attaches itself to an incompatible host, transfer cells specializing in water and nutrient uptake are initiated at the interface, but the transfer of nutrients via the phloem sieve tube does not occur [8]. Obviously, in both compatible and incompatible interactions, tight coordination of growth and differentiation between a parasite plant and its host is essential. It is challenging, however, to assign the underlying molecular events to specific cells belonging to *Cuscuta* or its host.

The formation of these cellular structures at the cell-to-cell interface seems to tighten the physical connection, thus making it difficult to detach cells of the parasite from host cells to investigate gene expression profiles of respective plants. Given that morphological markers exist, the individual tissues can be isolated using laser microdissection and subjected to RNA-Seq (whole-transcriptome shotgun sequencing) analysis [15]. Nevertheless, the *Cuscuta* tissue at the interface represents a highly complex branched structure composed of haustorial tissue and searching hyphae [16]. Thus, in most instances, this tissue is too complex to be dissected and analyzed in a simple fashion. An alternative method could be to classify RNA sequencing data using a bioinformatics approach. For instance, in transcriptomic analysis of *Cuscuta pentagona* using RNA-Seq (whole-transcriptome shotgun sequencing), reads originating from the host plant were removed using the reference sequences of compatible hosts [15,17,18]. In the analysis of RNA movement between *C. pentagona* and host plants (*Arabidopsis* and tomato), similar read classification based on the similarity to the host’s reference sequences was performed to distinguish transcripts from parasite plant and host plant [12]. Since complete genome sequences for *Cuscuta* spp. and their natural hosts are not available, the above classification and filtering cannot be used. However, the latest next-generation sequencing technology provides sufficient depth (numbers of reads) and sequence length to classify reads and to identify specific expression patterns.

In this study, we describe a bioinformatics approach to classify RNA-Seq reads obtained from an interface region formed between the non-model parasite plant *Cuscuta japonica* and the non-model host plant *Impatiens balsamina*. Without established reference sequences, we classified RNA-Seq reads using a stepwise classification procedure by means of similarity search to i) sequences obtained from tissues harvested from non-feeding *C. japonica* and *I. balsamina,* ii) sequences of plants belonging to the same genus, particularly of *C. reflexa* which is the phylogenetically closest species to *C. japonica* [4] and iii) sequences of the same family. The filtered sequences were used for *de novo* transcriptome assembly of tissues consisting of cells from *C. japonica* and *I. balsamina*. Using a competitive mapping approach in which reads were mapped to contigs of both plants, we quantitatively assessed the probability of misclassification. We achieved a misclassification rate low enough (0.22-0.39%) to avoid a significant difference of accuracy in identifying differentially expressed genes compared to conventional mapping. We then applied the read classification strategy to the RNA-Seq data from the interface between *C. japonica* and a model plant, *Glycine max* (soybean). RNA-Seq reads obtained from *C. japonica*-*G. max* interface regions at 5 parasitizing stages were subjected to the read classification, and genes regulated in a stage-dependent manner were identified both for *C. japonica* and *G. max*. The read classification method presented here will be useful for analyzing other multi-organism systems.

## Results and discussion

### Classifying RNA-Seq reads derived from two non-model plants

**Figure 1:**
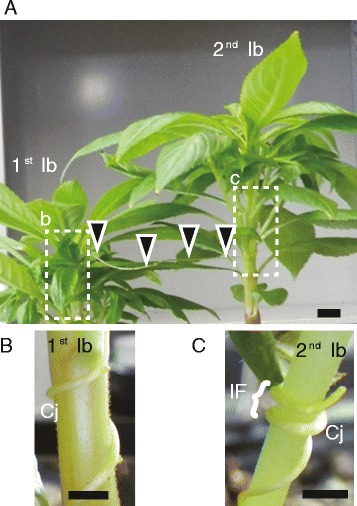
Experimental setup of *C. japonica*-*I. balsamina* association. **(A)** *C. japonica* (Cj) made parasite-host association to the first *I. balsamina* individual (1^st^ Ib, left). Elongated *C. japonica* attached to the second *I. balsamina* individual (2^nd^ Ib, right) and made parasite-host association. Interface region (IF) was sampled from the second *I. balsamina* (2^nd^ Ib, right). Black triangles indicate an elongated stem of *C. japonica*. **(B)** Magnified image of the interface region on the stem surface of the first *I. balsamina* (1^st^ Ib). **(C)** Magnified image of the interface region of the second *I. balsamina* (2^nd^ Ib). Tissue indicated with a white curly bracket (IF) was sampled as the interface region tissue. Scale bars indicate 1 cm.

**Figure 2:**
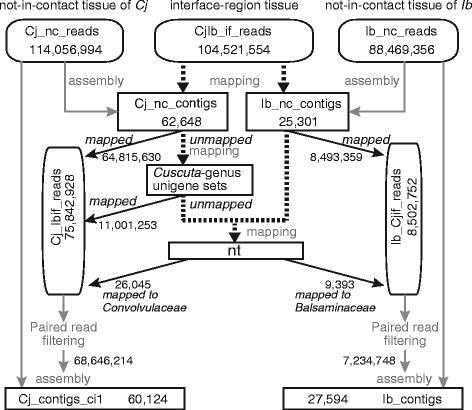
Schematic diagram of classification of reads. Dotted lines indicate that mapping was performed. Solid lines indicate that assembly or classification was performed. Abbreviations: Cj, *C. japonica*; Ib, *I. balsamina*; nc, not-in-contact; if, interface region. Numbers indicate the numbers of reads.

We attempted to identify genes expressed in the interface region formed between two non-model plants, the parasite *C. japonica*, and the host, *I. balsamina* (Figure 1). Our first aim was to assemble *de novo* transcriptome sets using RNA-Seq reads obtained from the interface region without physically dissecting either the parasite’s or host’s tissues. Instead of using physical dissection, we employed a bioinformatic approach to classify the reads into two groups; 1) *C. japonica*, and 2) *I. balsamina*. As references for classification of the reads, sequence sets of the transcriptome were assembled from samples of *C. japonica* and *I. balsamina* that grew independently and were not in contact (nc; Figure 2). Sets of RNA-Seq reads derived from *C. japonica* (Cj_nc_reads) and *I. balsamina* (Ib_nc_reads) can be considered non-contaminated. We assembled the reads from these sets into 62,648 Cj_nc_contigs and 25,301 Ib_nc_contigs, respectively. The difference in contig numbers could be due to the tissue specificity of the gene expression in the *C. japonica* subapical stem and the *I. balsamina* stem.

The reads obtained from the interface region (Figure 1C) in which *C. japonica* parasitized to *I. balsamina* (CjIb_if_reads; Figure 2) were classified using three sequential rounds of mapping. First, we mapped the CjIb_if_reads separately to the contigs from tissues that were not-in-contact, Cj_nc_contigs and Ib_nc_contigs. Reads that were uniquely mapped onto either Cj_nc_ contigs or Ib_nc_ contigs were classified as originating from *C. japonica* or *I. balsamina*, respectively (Figure 2). The reads that remained unmapped at this point likely contained transcripts specific to the interface region. To distinguish *C. japonica* reads from *I. balsamina* reads, we used previously published unigene sets of *C. pentagona* and *C. suaveolens* [17,18]. In addition, we constructed a novel contig set of *C. reflexa*, the species phylogenetically closest to *C. japonica* [4]. This analysis was performed using RNA-Seq reads from self-parasitizing tissue in which the subapical region of *C. reflexa* formed haustorial connections to distant parts of its own stem or to other *C. reflexa* individuals feeding on tomato. The *C. reflexa* transcriptome was assembled from 308,147,540 paired-end reads and consisted of 165,213 contigs (Cr_contigs, Table 1). We performed the second classification by mapping the reads that remained unmapped in the first classification onto these *Cuscuta*-genus unigene sets. The mapping reads were then considered as originating from *C. japonica* (11,001,253 reads). During the final step, we mapped the remaining reads to the NCBI nt (nucleotide) database. If reads mapped to nucleotide entries from Convolvulaceae or Balsaminaceae families, they were considered to be derived from *C. japonica* or *I. balsamina*, respectively. This step revealed additional 26,045 *C. japonica* and 9,393 *I. balsamina* reads.

**Table 1:** ***De novo*** **assembly and annotation of** ***C. reflexa*** **,** ***C. japonica,*** **and** ***I. balsamina*** **contigs**

	***C. reflexa*** **(Cr_contigs)**	***C. japonica*** **(Cj_contigs_ci1)**	***I. balsamina*** **(Ib_contigs)**
**Library type**	Illumina, 90 bp paired-end	Illumina, 101 bp paired-end	Illumina, 101 bp paired-end
**Assembler**	Trinity	Velvet/Oases	Velvet/Oases
**Assembled reads**	308,147,540	182,703,208	95,704,104
**Number of contigs**	165,213	60,124	27,594
**Median contig length**	377 bp	599 bp	1,026 bp
**Average contig length**	620 bp	980 bp	1,276 bp
**ORF predicted**	89,456 (54%)	59,768 (99%)	27,507 (99%)
**Full-length ORF**	64,442 (39%)	33,313 (55%)	18,118 (66%)

After this stepwise classification, 73% (75,842,928 reads) of the sequence information from the interface region (104,521,554 CjIb_if_reads) could be mapped to *C. japonica*, whereas 8.2% (8,502,752 reads) mapped to *I. balsamina* (Figure 2). This difference in the numbers of classified reads was probably due to difference in the concentration of RNA in the parasite and the host tissues. The amount of total RNA per mg fresh weight of not-in-contact stem tissue of *C. japonica* was 16.8 ± 6.5-fold greater than that in an *I. balsamina* sample of equal mass (Additional file 1). Thus, in the interface-region samples that contained nearly equal amounts of parasite and host tissues, a larger number of *C. japonica* reads was expected. In a previous study on the associations between *C. pentagona* and *Arabidopsis* and *C. pentagona* and tomato, the interface regions contained higher portions of host reads (51% from *Arabidopsis* and 86% from tomato) [12]. The discrepancy with the present study might be due to the fact that the interface region in this study was sampled from the stem of *C. japonica* closer to the apex (1 cm from the tip) than the region used in the *C. pentagona* study (>7.5 cm from the tip) [12], and therefore resulted in a higher percentage of *C. japonica* reads.

### *De novo* assembly and annotation

The classified reads from the interface region (Cj_Ibif_reads and Ib_Cjif_reads) were merged with the respective read set from not-in-contact tissues (Cj_nc_reads or Ib_nc_reads). The merged read sets were used for *de novo* transcriptome assembly using Velvet/Oases [19,20]. The assembled C*. japonica* and *I. balsamina* transcriptomes consisted of 60,124 contigs (Cj_contigs_ci1, median length 599 bp, average length 980 bp; Table 1) and 27,594 contigs (Ib_contigs, median length 1,026 bp, average length 1,276 bp; Table 1).

The *C. reflexa* transcriptome used in read classification was based on 308,147,540 paired-end reads as input for *de novo* assembly using Trinity (version r20140717) [21]. The raw assembly consisted of 165,213 Cr_contigs (246,886 transcript variations, with median length 377 bp, average length 620 bp; Table 1). We could predict ORFs in 89,456 *C. reflexa* contigs (median length 330 bp). Of these contigs, 64,442 contained full-length ORFs (median length 363 bp).

These numbers of assembled transcripts may be an overestimation for the actual numbers of genes expressed in the tissues. This overestimation is possibly due to the presence of alleles, alternatively spliced transcripts, and fragmented transcripts. Further refinement will be required on the basis of annotation.

The gene ontology (GO) category distributions for Cj_contigs_ci1 and Ib_contigs, based on the similarity to the RefSeq database [22] and TAIR10 [23], did not have a bias toward any specific categories (Additional file 2). A similarity search for transcripts of other plants revealed that *C. japonica* transcripts showed the highest similarity to *C. reflexa*, and showed lower similarity to parasitic plants belonging to the Orobanchaceae family (Additional file 3).

### Quality assessment of read classification

The transcripts of *C. japonica* and *I. balsamina* were *de novo* assembled from all reads including those obtained from the interface region. This fact prompted us to estimate the extent of misclassification of *C. japonica* reads as *I. balsamina* transcripts or vice versa. To this end, we performed a competitive mapping. In conventional cases, RNA-Seq reads obtained from an organism are mapped solely onto the reference sequence of that organism. Here, our strategy was to map reads onto both *C. japonica* and *I. balsamina* contigs. A binary choice was made based on a higher mapping score (identity and *e* value) in order to assign reads to one of the two species. Since using reads from the interface-region samples would make it harder to properly discriminate the true and false source organisms, we used only Cj_nc_reads and Ib_nc_reads for our estimation.

**Figure 3:**
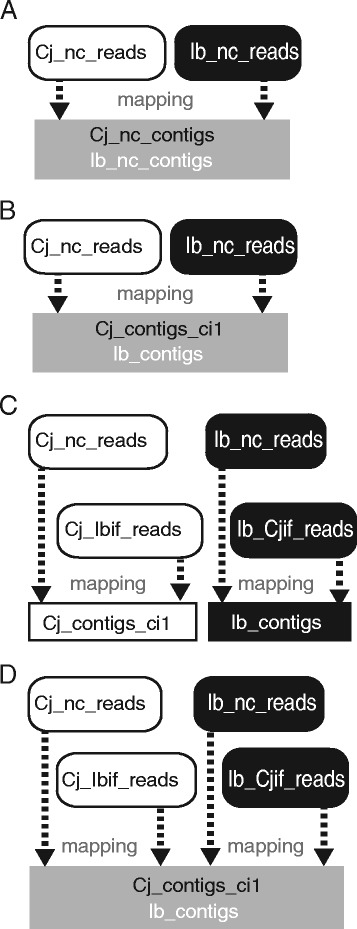
Scheme of read mapping to estimate the rate of false classification. **(A)** Cj_nc_reads and Ib_nc_reads were mapped to a merged transcript set of Cj_nc_contigs and Ib_nc_contigs. **(B)** Cj_nc_reads and Ib_nc_reads were mapped to a merged transcript set of Cj_contigs_ci1 and Ib_contigs. **(C)** Cj_nc_reads and Cj_Ibif_reads were mapped onto Cj_contigs_ci1. Ib_nc_reads and Ib_Cjif_reads were mapped onto Ib_contigs. **(D)** Cj_nc_reads, Cj_Ibif_reads, Ib_nc_reads and Ib_Cjif_reads were mapped onto a merged transcript set of Cj_contigs_ci1 and Ib_contigs. The result of the mapping in panel **(C)** was used for the identification of differentially expressed genes.

Misclassification during mapping will inevitably occur due to the presence of homologous transcripts between *C. japonica* and *I. balsamina* (Additional file 3). To estimate the extent to which this misclassification can be attributed to the presence of homologous transcripts, we mapped Cj_nc_reads and Ib_nc_reads to a merged dataset consisting of Cj_nc_contigs and Ib_nc_contigs (Figure 3A). Because Cj_nc_contigs and Ib_nc_contigs were assembled separately from the two nonoverlapping read sets, any instance of misclassification had to occur due to the presence of identical sequences in the parasite and the host. According to this test, the background rates of false classification were revealed as: 2.38% (Cj_nc_reads mapped to Ib_nc_contigs) and 0.22% (Ib_nc_reads mapped to Cj_nc_contigs; Table 2A).

**Table 2:** **Assessment for the rate of misclassification of reads by mapping Cj_nc_reads and Ib_nc_reads onto two transcript sets**

A.	Contig set	Merged set of Cj_nc_contigs and Ib_nc_contigs		
	Classification	Reads mapped onto *C. japonica* (%)^a^	Reads mapped onto *I.balsamina* (%)^a^	AUC^b^
	Mapped reads	Number of reads (%)		0.981
	Cj_nc_reads	76,775,986	2,712,459	
		(67.3)	(2.38)	
	Ib_nc_reads	195,363	71,599,224	
		(0.22)	(80.9)	
B.	Contig set	Merged set of Cj_contigs_ci1 and Ib_contigs		
	Classification	Reads mapped onto *C. japonica* (%)^a^	Reads mapped onto *I.balsamina* (%)^a^	AUC^b^
	Mapped reads	Number of reads (%)		0.995
	Cj_nc_reads	73,819,654	442,526	
		(64.7)	(0.39)	
	Ib_nc_reads	271,929	71,471,262	
		(0.31)	(80.8)	
C.	Contig set	Merged set of Cj_contigs_ci1 and Ib_contigs		
	Classification	Reads mapped onto *C. japonica* (%)^a^	Reads mapped onto *I.balsamina* (%)^a^	AUC^b^
	Mapped reads	Number of reads (%)		0.995
	Cj_nc_reads	58,501,463	324,744	
		(55.3)	(0.28)	
	Ib_nc_reads	197,487	60,709,899	
		(0.22)	(68.6)	

^a^Numbers indicate percentage of mapped reads to the total number of resds. ^b^AUC; area under receiver operating characteristic (ROC) curve.

Mapping parameter was as follows; In Panel A and Panel B, match length ≥90 bp and at most 1 mismatch and 1 gap allowed. In Panel C, match length =100 bp and no mismatch allowed.

In comparison with this mapping, we mapped Cj_nc_reads and Ib_nc_reads to a merged transcript set consisting of Cj_contigs_ci1 and Ib_contigs (Figure 3B). During this round of mapping, an instance of false assignment could be attributed to read misclassification that occurred prior to the assembly of Cj_contigs_ci1 and Ib_contigs, in addition to transcript homology. Here, 0.39% of Cj_nc_reads were mapped to Ib_contigs and 0.31% of Ib_nc_reads were mapped to Cj_contigs_ci1 (Table 2B), suggesting that these cross-mapped reads were misclassified. We estimated the rate of false classification under more stringent conditions where only exact matches were allowed. The frequency of false assignments of Cj_nc_reads to Ib_contigs decreased to 0.28% and that of Ib_nc_reads to Cj_contigs_ci1 decreased to 0.22% (Table 2C).

Finally, we evaluated the quality of the classification by analyzing the receiver operating characteristic (ROC) and its area under the curve (AUC) [24]. The AUC approaches 1.0 when better classification is achieved with a greater ratio of true positive to false positive results. When nc_reads were mapped onto nc_contigs, the AUC was 0.981 (Table 2A). When nc_reads were mapped onto contigs assembled from classified reads, we obtained a higher AUC of 0.995 (Table 2B).

These results collectively demonstrated that, when we mapped the reads obtained from the interface region to the transcripts assembled from them, these reads could be assigned to the wrong plant. Nevertheless, according to the ROC AUC, read classification performed prior to transcript assembly did not impair the binary choice more strongly than in the case of false assignments solely due to the presence of homologous transcripts. These results raise the question whether false assignments can happen more frequently between plant species that are more closely related. A systematic analysis of RNA-Seq reads from many plant species will be necessary to find an answer to this question.

We next tested whether or not including misclassified reads in the assembly of Cj_contigs_ci1 can lead to significantly different results with respect to the identification of differentially expressed genes between not-in-contact and interface-region tissues. To test this idea, we analyzed two different mapping results to identify differentially expressed genes (Additional file 4). First, Cj_nc_reads and Cj_Ibif_reads were separately mapped to Cj_contigs_ci1. Differentially expressed genes were then identified by comparison of the two mapping results. The same procedure was applied to Ib_nc_reads and Ib_Cjif_reads by separately mapping them onto Ib_contigs (Figure 3C). Second, to exclude potentially misclassified reads from the estimation of gene expression, Cj_nc_reads and Cj_Ibif_reads were separately mapped to a transcript set containing both Cj_contigs_ci1 and Ib_contigs (Figure 3D). Ib_nc_reads and Ib_Cjif_reads were also separately mapped to the same merged contig set (Figure 3D). Here, reads that were mapped to the wrong set (*C. japonica* reads to *I. balsamina* contigs or vice versa) were excluded from the RPKM-estimation. Both approaches yielded identical estimates for the number of differentially regulated transcripts in the interface region, except for the number of down-regulated genes in *C. japonica* (Table 3). This result implies that excluding potentially misclassified reads makes no significant difference for the identification of transcripts differentially expressed in the interface region.

**Table 3:** **Assessment of the accuracy in identifying differentially expressed genes in parasitic tissue**

**Mapping procedure**	**Separately onto Cj_contigs_ci1 and Ib_contigs** ^**c**^	**To a merged set of Cj_contigs_ci1 and Ib_contigs** ^**d**^
**Differential expression**	**number of contigs**	**number of contigs**
Upregulated in *C. japonica*	284^a^	284^a^
Downregulated in *C. japonica*	944^b^	940^b^
Upregulated in *I. balsamina*	10^a^	10^a^
Downregulated in *I. balsamina*	530^a^	530^a^

^a^ Member contigs are the same in all classes. ^b^ 939 transcripts were found in common. ^c^ Corresponds to Figure 3C. ^d^ Corresponds to Figure 3D.

### Application of the read classification to *C. japonica*-model plant interaction

**Figure 4:**
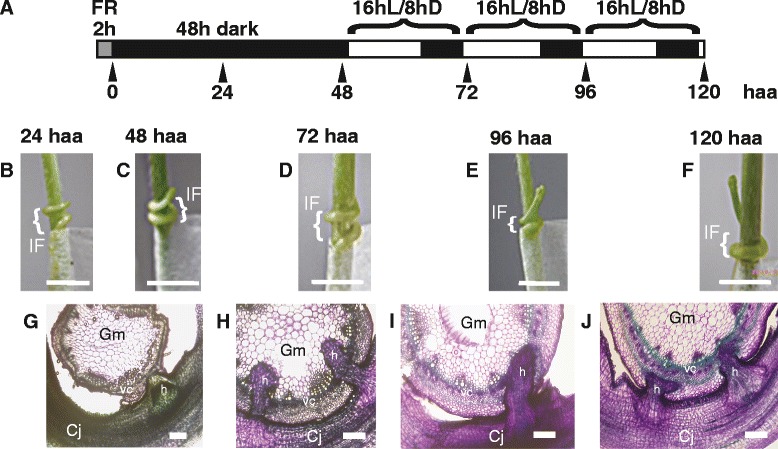
Experimental setup of *C. japonica*-*G. max* association. **(A)** A sampling schedule of the interface tissues at five parasitizing stages. Light and dark conditions are indicated by white and black areas, respectively. Just after attaching *C. japonica* to *G. max* stem, a *G. max* plant with *C. japonica* attached to its stem was placed under far red (FR) illumination and kept for 2 h. From 0 hour after attachment (haa) to 48 haa, a *G. max* plant with *C. japonica* attached to its stem was grown in the continuous dark [30]. The 48 haa-sample was harvested in the dark. From 48 haa to 120 haa, plants were grown in the 16 h/8 h light/dark condition. Black triangles indicate the time points of sampling. **(B-F)** Appearance of the interface region on the stem surface of *G. max*. IF, interface region; scale bars, 1 cm. **(G-J)** Cross sections of the interface region. Gm, *G. max* tissue; Cj, *C. japonica* tissue; vc, vascular cambium of *G. max*; h, haustorium of *C. japonica*. Scale bar, 10 μm. At 24 haa, a section of the interface region was not successfully made due to the weak adhesion of *C. japonica* to *G. max*. Note that, in panel H, haustorium (h) of *C. japonica* appears to penetrate the vascular cambium (vc) of *G. max* and the tip of haustorium reached the pith.

**Figure 5:**
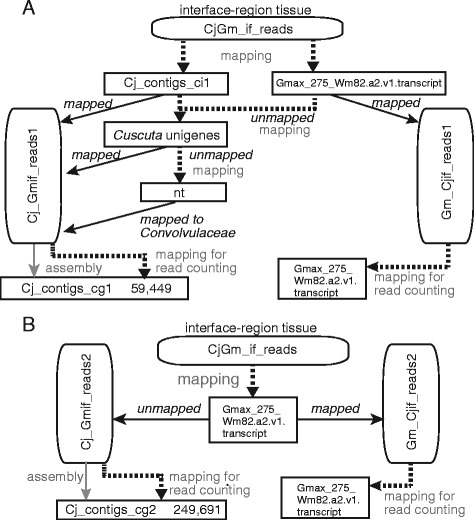
Schematic diagram of classification of reads from *C. japonica*-*G. max* interface region. Dotted lines indicate that mapping was performed. Solid lines indicate that assembly or classification was performed. Abbreviations: Cj, *C. japonica*; Gm, *G. max*; nc, not-in-contact; if, interface. **(A)** Stepwise classification. **(B)** Reference-based classification.

We applied the read classification method to RNA-Seq data obtained from the interface region between *C. japonica* and a model-plant host, *Glycine max* (soybean) whose reference transcriptome was available [25] (Figure 4). Interface regions were sampled from five parasitizing stages at 24 h after attachment (haa), 48 haa, 72 haa, 96 haa and 120 haa (Figure 4A). Reads from interface regions were classified using two approaches. The “stepwise classification” approach (Figure 5A) was based on the reference transcriptome of *G. max*, Gmax_275_Wm82.a2.v1.transcript [26] to select *G. max* reads, and used the read classification method presented in the previous sections to identify *C. japonica* reads. By contrast, the “reference-based-classification” approach (Figure 5B), mapped reads from the interface regions onto a reference transcriptome set of *G. max*. Reads that mapped to the reference were regarded as *G. max* reads, non-mapping reads were assumed to originate from *C. japonica*.

*C. japonica* reads from the five parasitizing stages were pooled and subjected to *de novo* transcriptome assembly. Reads obtained by stepwise classification were assembled into 59,449 contigs (Cj_contigs_cg1, Table 4). On the other hand, reads obtained by reference-based classification were assembled into 249,621 contigs (Cj_contigs_cg2, Table 4). A larger number of short contigs (i.e. <200 bp) that did not have similarity to the RefSeq entries was present in Cj_contigs_cg2 than Cj_contigs_cg1 (Table 4). This result implied that the quality of the contig set obtained by stepwise classification was better than that obtained by reference-based classification.

**Table 4:** ***De novo*** **assembly and annotation of** ***C. japonica*** **contigs classified in two different approaches**

	**Cj_contigs_cg1**	**Cj_contigs_cg2**
Library type	Illumina, 74 bp single-end	Illumina, 74 bp single-end
Assembler	Velvet/Oases	Velvet/Oases
Assembled reads	141,089,611	205,957,611
Number of contigs	59,449	249,621
Median contig length	325 bp	187 bp
Average contig length	648 bp	354 bp
Length distribution (%)		
<200 bp	18,009 (30.3)	134,968 (54.1)
201-500 bp	19,803 (33.3)	75,199 (30.1)
501-1000 bp	9,739 (16.4)	22,305 (8.9)
>1001 bp	11,898 (20.0)	17,149 (6.9)
Length distribution of contigs no-hits-found in refseqplant (%)		
<200 bp	13,986 (23.5)	114,864 (46.0)
201-500 bp	12,232 (20.6)	53,096 (21.3)
501-1000 bp	3,160 (5.3)	10,616 (4.3)
>1001 bp	952 (1.6)	2,639 (1.1)

Next, we compared the probability of misclassification between the two approaches. We performed a competitive mapping of reads obtained from not-in-contact stems, Cj_nc_reads and Gm_nc_reads, separately against merged contig sets consisting of the *G. max* reference transcriptome and either Cj_contigs_cg1 or Cj_contigs_cg2. Using Cj_contigs_cg1 resulted in a higher AUC value (0.999; Table 5A) than using Cj_contigs_cg2 (0.955; Table 5B), suggesting that the stepwise classification approach is better.

**Table 5:** **Assessment for the rate of misclassification of reads by mapping Cj_nc_reads and Gm_nc_reads onto two contig sets**

A.	Contig set	Merged set of Cj_contigs_cg1 and Gmax_275_Wm82.a2.v1.transcript		
	Classification	Reads mapped onto *C. japonica* (%)^a^	Reads mapped onto *G.max* (%)^a^	AUC^b^
	Mapped reads (number)	Number of reads (%))		
	Cj_nc_reads	54,936,999	187	0.999
	(114,056,994)	(48.2)	(0.00016)	
	Gm_nc_reads	1,791	25,880,696	
	(28,728,782)	(0.0062)	(80.9)	
B.	Contig set	Merged set of Cj_contigs_cg2 and Gmax_275_Wm82.a2.v1.transcript		
	Classification	Reads mapped onto *C. japonica* (%)^a^	Reads mapped onto *G.max* (%)^a^	AUC^b^
	Mapped reads (number)	Number of reads (%)		
	Cj_nc_reads	52,486,992	191	0.955
	(114,056,994)	(46.0)	(0.00017)	
	Gm_nc_reads	2,377,249	24,253,863	
	(28,728,782)	(8.27)	(84.4)	

^a^Numbers indicate percentage of mapped reads to the total number of resds. ^b^AUC; area under receiver operating characteristic (ROC) curve.

Mapping parameter was as follows; In Panel A and Panel B, match length ≥90 bp and at most 1 mismatch and 1 gap allowed.

For the detection of differentially expressed genes during establishment of parasitic connection, the Cj_contigs_cg1 provided a more robust result than the Cj_contigs_cg2 (Table 6). We compared the number of detected differentially expressed genes by either i) mapping reads classified from the interface region (Cj_Gmif_reads and Gm_Cjif_reads) separately to the *C. japonica* and *G. max* contig sets, or ii) by mapping Cj_Gmif_reads and Gm_Cjif_reads separately to a merged contig set consisting of *C. japonica* contigs and *G. max* contigs. When combining Cj_contigs_cg1 with the *G. max* reference transcriptome, the differences in numbers of differentially expressed genes between the two cases (17,653 and 17,656; Table 6) were smaller than when combining Cj_contig_cg2 with the *G. max* reference transcriptome (17,653 and 17,526; Table 6). Collectively, these results confirmed that the stepwise classification approach resulted in a better *de novo* transcriptome assembly, at least in the case of *C. japonica*, with respect to the quality of the contig set and robustness in the identification of differentially expressed genes.

**Table 6:** **Assessment of the accuracy in identifying differentially expressed genes in interface regions of** ***C. japonica*** **-** ***G. max*** **association**

*C.japonica* read	Cj_Gmif_reads1		Cj_Gmif_reads2	
*G. max* read	Gm_Cjif_reads1		Gm_Cjif_reads2	
*C. japonica* contig	Cj_contigs_cg1		Cj_contigs_cg2	
*G. max* contig	Gmax_275_Wm82.a2.v1.transcript		Gmax_275_Wm82.a2.v1.transcript	
Mapping procedure	Separately^a^	Merged^b^	Separately^a^	Merged^b^
Differentially expressed genes^c^ in *C. japonica* in *G. max*	number of contigs (%)^d^		number of contigs (%)^d^	
	3,819	3,819	10,806	10,806
	(6.4)	(6.4)	(4.3)	(4.3)
	17,653	17,656	17,653	17,526
	(19.9)	(19.9)	(19.9)	(19.7)

^a^Indicated reads were mapped onto *C. japonica* contig and *G. max* contig separately, and uniquely hit reads to each contig set was used to estimate gene expression level. ^b^Indicated reads were mapped onto a merged contig set of *C. japonica* contig and *G. max* contig. If a given read hit to wrong contigs (*C. japonica* read to *G. max* contig, or vice versa) that read was excluded from the estimation of gene expression level. ^c^Differentially expressed genes detected by using TCC software [45] with the q-value <0.05. ^d^Percentage of the number of differentially expressed genes to the total number of *C. japonica* or *G. max* contigs.

### Comparative gene expression profiling of the parasite plant and the host plant

**Figure 6:**
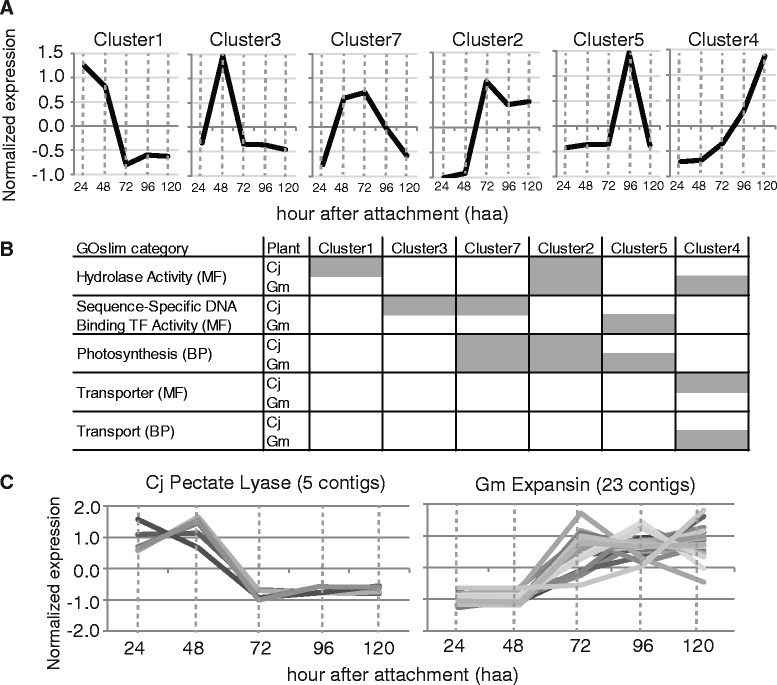
Cluster analysis of differentially expressed genes in the interface region of *C. japonica*-*G. max* association. **(A)** Expression patterns of clusters that had single expression peak at one of the 5 stages. Both cluster 7 and cluster 2 peaked at 72 haa. **(B)** GOslim categories enriched in *C. japonica* and *G. max*. Grey areas indicate occurrence of GOslim category enrichment. Abbreviations: MF, molecular functions; CC, cellular components; BP, biological processes; TF, transcription factor. **(C)** Expression patterns of 5 *C. japonica* contigs with similarity to pectate lyase (*e* value < 1e-10) and 23 *G. max* contigs with similarity to *LeEXPA5* (*e* value < 1e-10).

Distinguishing read origins via classification allowed us to simultaneously monitor gene expression profiles of both *C. japonica* and *G. max* in the interface region. We used Cj_contigs_cg1 together with the *G.* max reference transcriptome for mapping reads from the interface region, and identified differentially expressed transcripts (Additional file 5). Expression profiles of all differentially expressed transcripts, 3,819 *C. japonica* contigs and 17,653 *G. max* contigs, were collectively subjected to cluster analysis and classified into 10 clusters according to the expression patterns across the stages (Table 7). Each parasitizing stage was characterized as follows. At 24 haa, *C. japonica* had completed coiling around *G. max* stem, and prehaustorium structures formed (Figure 4B). At 48 haa, elongation of *C. japonica* was arrested, and the tip of the haustorium was localized in the cortex (Figure 4C and G). At 72 haa, elongation of *C.japonica* was still arrested, and the tip of the haustorium was localized in the pith (Figure 4D and H). At 96 haa, elongation of *C.japonica* restarted, and the tip of the haustorium was localized at the vascular cambium (Figure 4E and I). At 120 haa, the stem of *C. japonica* elongated, and and the tip of the haustorium still localized at the vascular cambium (Figure 4F and J). We focus here on the 6 clusters (cluster1, cluster 3, cluster 7, cluster 2, cluster 5 and cluster 4) whose expression profile peaked at one of the 5 stages (Figure 6A).

**Table 7:** **Number of differentially expressed genes in each cluster**

**Cluster**	**Number of differentially expressed genes (%)**				**Description**
	***C. japonica***		***G. max***		
1	1,666	(43.6)	4,940	(28.0)	max. at 24 haa
2	1,024	(26.8)	8,828	(50.0)	max. at 72 haa
3	326	(8.5)	489	(2.8)	max. at 48 haa
4	355	(9.3)	2,866	(16.2)	max. at 120 haa
5	68	(1.8)	67	(0.4)	max. at 96 haa
6	99	(2.6)	82	(0.5)	max. at 72 haa, min. at 48 haa
7	184	(4.8)	222	(1.2)	max. at 72 haa
8	63	(1.6)	127	(0.7)	max. at 24 haa, min. at 72 haa
9	23	(0.6)	18	(0.16)	max. at 72 haa, min. at 48 haa
10	11	(0.28)	4	(0.02)	max. at 72 haa, min. at 96 haa

In several GOslim categories enriched in these stages, we found similarities as well as differences in the composition of underlying transcripts between *C. japonica* and *G. max* (Figure 6B). Under the GOslim category of “Hydrolase Activity”, transcripts encoding carbohydrate-, lipid- and protein-degrading enzymes were consistently found in cluster 1 and cluster 2 of *C. japonica*. On the other hand, in cluster 2 of *G. max*, the number of contigs related to the ubiquitin-dependent protein catabolic process increased transiently. In cluster 4 of *G. max*, the number of contigs associated with defense response increased, i.e., disease resistance protein (TIR-NBS-LRR class) family. A fact that approximately 44% of *C. japonica* contigs and 36% of *G. max* contigs in this category were associated with “Extracellular Region”, “Cell Wall” and “Plasma Membrane” reinforced the notion that various molecular interactions occur in the extracellular region between parasite and host. Expression of ubiquitin-proteasome pathway genes can be regarded as a part of this response, although it is not clear whether the ubiquitin-proteasome pathway plays an active role in terms of defense.

Enrichment of the GOslim category “Sequence-Specific DNA Binding Transcription Factor Activity” was observed earlier in *C. japonica* (cluster 3 and cluster 7) and later in *G. max* (cluster 5), suggesting that *C. japonica* led the initiation of cellular changes in the interface region. Twenty-seven *C. japonica*- and six *G. max* contigs were included in this category. Among them, Cj_contigs1.4_05837_00791 and Glyma.02G066200.1 exhibited similarity to the *ERF BUD ENHANCER* (*EBE*) gene, which positively regulates cell proliferation [27]. This observation tempted us to hypothesize that *EBE* plays distinct roles in the parasite and host plants at different stages of parasitism.

Enrichment of the GOslim category “Photosynthesis” was an inevitable consequence of our experimental setting in which plants were transferred from continuous dark to the light/dark cycle after 48 haa. The GOslim categories of “Transporter Activity” and “Transport” were enriched in cluster 4 of *C. japonica* and *G. max,* respectively, and up-regulation of various transporter genes was observed. Increase in the transcriptional activity of transporter genes probably coincided with the increase in sink activity of *C. japonica*.

Modification of the cell wall is one of the key processes in establishing a cellular connection between parasite and host. Expansin belongs to the group of cell wall modifying proteins responsible for cell wall extension under acidic conditions. Tomato expansin gene, *LeEXPA5*, has been reported to be upregulated in root during syncytia formation by potato cyst nematode [28]. Cell wall disassembly by nematode triggers expression of host cell wall modifying proteins. To test whether a plant parasite also triggers expression of the host’s cell wall modifying proteins, we investigated the temporal difference between the genes encoding the parasite’s pectate lyase, a cell wall degrading enzyme, and the host’s expansin, a cell wall modification protein. Expression levels of 5 *C. japonica* contigs that exhibited similarity to pectate lyase (*e* value < 1e-10) peaked at 24 haa or 48 haa and then decreased (Figure 6C, left). We identified 23 *G. max* contigs that exhibited similarity to *LeEXPA5* (*e* value < 1e-10). The expression levels of these contigs peaked at 72-, 96- or 120-haa (Figure 6C, right). These results demonstrated that the expression of cell wall degrading enzyme genes in *C. japonica* preceded the expression of expansins in *G. max*. Simultaneous profiling of gene expression in both parasite and host allowed us to monitor the temporal differences, and helped inferring the coordination or causal relationship between cellular processes in the two plants.

## Conclusion

We demonstrated that simultaneous analysis of gene expression profiles in a non-model parasitic plant, *C. japonica*, and a non-model host plant, *I. balsamina*, can be performed using RNA-Seq reads obtained from an interface region containing cells of both plants. We performed stepwise classification of reads using sequences of the plant species under study, plants belonging to the same genus, and, finally, plants in the same family. Using reads classified in this way, we *de novo* assembled the transcriptome sequence sets of the interface region. To confirm the annotation, we also assembled the transcriptome of *C. reflexa*. Applying a competitive mapping method, we could assess the quality of the performed classification. This assessment revealed that we achieved classification of reads with a misclassification rate low enough to be used reliably for analysis of differential expression of genes. We applied this read classification method to simultaneously analyze gene expression profiles in the non-model parasitic plant *C. japonica* and the model host plant *G. max*. We assembled the *C. japonica* transcriptome from reads classified by our stepwise classification approach. Using our *C. japonica* transcriptome in combination with the *G. max* reference transcriptome, we were able to robustly identify differentially expressed genes in both parasite and host. This simultaneous monitoring of gene expression in both parasitic and host plants shed new lights on coordination of cellular processes between two plants. This approach may be applicable to other multi-organism systems.

## Methods

### Plant materials

*I. balsamina* was grown on soil (Sukoyaka-Baido, Yanmar Co. Ltd., Osaka, Japan) mixed with the same volume of vermiculite (GS30L, Nittai, Osaka, Japan) in a 16 h/8 h light/dark cycle at 25 °C. *C. japonica* seeds were dipped in concentrated sulfuric acid for 15 min, washed with distilled water and plated on wet glass filter paper (GA-100, Toyo Roshi Kaisha, Ltd., Tokyo, Japan) in the dark at 25 °C. Parasitism was induced by attaching the subapical region of *C. japonica* to the stem of *I. balsamina* and illuminating the junction with far red light (FL20S・FR-74, Toshiba, Tokyo, Japan) for 2 h [29]. After the far-red-light illumination, the plants were kept in darkness 48 h [30].

The interface region containing both *C. japonica* and *I. balsamina* tissues was harvested from the secondary *C. japonica*-*I. balsamina* association (Figure 1). A subapical region, 1–2 cm below the apical tip, of a 7-day-old *C. japonica* seedling grown in vermiculite (GS30L, Nittai, Osaka, Japan) in a 16 h/8 h light/dark cycle at 25 °C was attached to the stem of the first 40-day-old *I. balsamina*, and parasitism was induced as described above. After 14 days, the subapical region extending from the first interface region was attached to the stem of the second 40-day-old *I. balsamina*, and parasitized as described above. The material from the secondary interface region containing both *C. japonica* and *I. balsamina* tissues was sampled after the 24 h-dark treatment in the middle of 48 h-dark treatment [30]. In order to obtain not-in-contact (nc) *C. japonica* samples, the sub-apical region of an 8- to 10-day-old *C. japonica* was attached to a plastic rod (diameter 5 mm) and subjected to the far-red light treatment and subsequent dark treatment. Subsequently, subapical region, 1–2 cm below the apical tip, was harvested. To obtain not-in-contact *I. balsamina* tissue*,* the stem of a 40-day-old *I. balsamina* was coiled two turns using a plastic-coated wire. Stem was harvested after the far-red light treatment and subsequent darkness treatment.

Soybean (*Glycine max* cv. Fukuyutaka) was sown on soil (Sukoyaka-Baido, Yanmar Co. Ltd.) mixed with the same volume of vermiculite (GS30L, Nittai) and grown in a 16 h/8 h light/dark cycle at 25 °C. A 14-day-old *G. max* was parasitized by a 8- to 10-day-old *C. japonica* at the stem part between cotyledon and the first foliage leaf. Parasitism was induced as described above (Figure 4A). The interface region containing both *C. japonica* and *G. max* tissues was harvested at five stages, 24 hours after attachment (haa), 48 haa, 72 haa, 96 haa and 120 haa. Three replicates were prepared for each stage.

Tomato plants (*Solanum lycopersicum,* cv. Moneymaker) serving as a host for *C. reflexa* were grown under greenhouse conditions (relative humidity 55%, day temperature 25 °C, night temperature 20 °C, diurnal cycle: 16 h light/8 h darkness, and light intensity 190–600 μE · m^−2^ · s^−1^). The *C. reflexa* plants feeding on tomato stems were cut (~30 cm below the apex), transferred onto adult tomato stems, sprayed with water every 2 days, and covered with a plastic bag to facilitate the formation of haustorial connections. *C. reflexa* stems were harvested from *C. reflexa* individuals feeding on themselves or other individuals >30 cm away from the nearest haustorial connection to a tomato host plant. All tissues were harvested with sterile razor blades, immediately frozen in liquid nitrogen, and stored at −80 °C.

### RNA extraction, preparation of the sequencing library, and RNA-Seq

Total RNA extraction was performed using the Qiagen RNeasy Plant Kit (cat. # 74193, Qiagen, Netherlands) according to the manufacturer’s protocol. RNA integrity was confirmed using the Agilent 2100 BioAnalyzer (Agilent Technologies, Santa Clara, CA, USA). In experiments using *C. japonica* and *I. balsamina*, RNA-Seq libraries were prepared using Illumina’s TruSeq RNA Sample Prep Kit (RS-122-2001, RS-122-2002, Illumina Inc. San Diego, CA, USA) according to the manufacturer’s standard protocol. Three libraries were sequenced in one full lane on the Illumina HiSeq 2000 platform, and 101-bp paired-end reads were obtained from Hokkaido System Science Co., Ltd (Sapporo, Japan). The data on *C. japonica* and *I. balsamina* reads were registered in DNA Data Bank of Japan (DDBJ) Read Archive (http://trace.ddbj.nig.ac.jp/dra/) [31] [DRA:DRR021687, DRR021688 and DRR021689 in DRA002408]. RNA-Seq library of not-in-contact tissue of *G. max* was prepared by the same procedure.

In experiments using *C. japonica* and *G. max*, RNA-Seq libraries were prepared using NEBNext® Ultra™ RNA Library Prep Kit for Illumina® and NEBNext® Poly(A) mRNA Magnetic Isolation Module (E7490S, E7530S, Illumina Inc.). Each library was sequenced on the Genome Analyzer II (Illumina Inc.) in one full lane to yield 74 bp single-end reads. The data on *C. japonica* and *G. max* reads were registered in DDBJ Read Archive (http://trace.ddbj.nig.ac.jp/dra/) [31] [DRA: DRR030860, DRR030861, DRR030862, DRR030863 and DRR030864].

Total RNA extraction from *C. reflexa* samples was performed by grinding cut plant material in liquid nitrogen with immediate addition of the TRIzol Reagent (Invitrogen, Carlsbad, CA, USA; 0.5 mL per 100 mg tissue) as described previously [32]. After centrifugation (10,000 × g, 10 min, at 4 °C), the supernatant was transferred to a new tube, and an equal volume of phenol:chloroform:isoamyl alcohol (25:24:1, pH8.0; Roche, Basel, Switzerland) was added along with 1 μl RNasin (Promega, Fitchburg, WI, USA). The mixture was centrifuged at 10,000 × g for 10 min at 4 °C. The resulting supernatant was transferred to a new RNase-free plastic tube and extracted once with 200 μL and once with 50 μL of chloroform. To precipitate the total RNA, the supernatant was mixed with 2 volumes of 100% isopropanol, ^1^/_10_ volume of 3 M sodium acetate (pH 5.2), and 1 μg of linear acrylamide (Invitrogen), and the mixture was incubated for >1 h at −20 °C. After centrifugation (16,000 × g, 30 min, at 4 °C), the resulting pellet was washed twice with 80% ethanol, once with 99% ethanol, air dried, and resuspended in 20 μL of RNase-free water. To determine RNA quality and concentration, 1 μL of the RNA samples was subjected to agarose gel electrophoreses (2% agarose, 1× Tris-borate-EDTA [TBE] buffer) and was quantified using a NanoDrop device (Thermo Fisher Scientific, Waltham, MA, USA). The libraries were sequenced on the Illumina HiSeq 2000 platform, and 90-bp paired-end reads were obtained from the Beijing Genomics Institute (BGI; Shenzhen, China). The data of *C. reflexa* reads were registered in the NCBI Sequence Read Archive (http://www.ncbi.nlm.nih.gov/Traces/sra) [33] [SRA: SRR1171084].

### Preprocessing of raw reads

Read sets obtained from not-in-contact tissues of *C. japonica*, *I. balsamina*, and the interface region tissues were subjected to adapter removal, and to quality filtering using CASAVA ver.1.8.1 (Illumina) [34]. The read sets were filtered against a dataset of plant transfer RNA and ribosomal RNA sequences obtained from GenBank (gbpln[1–63].seq.gz, September 30, 2013) [35]. Reads that could be matched (*e* value ≤1e-5) to this in-house dataset using BLASTN [36] were removed. Furthermore, read pairs spanning <175 bp were also removed. This procedure yielded 3 read sets (Cj_nc_reads, Ib_nc_reads and CjIb_if_reads; Figure 1).

Read sets obtained from the interface regions of *C. japonica* and *G. max* were subjected to adapter removal, and to quality filtering using CASAVA ver.1.8.2 (Illumina). The read sets were filtered against the transfer RNA and ribosomal RNA sequences as above. This procedure yielded read sets of the five stages (CjGm_if_reads; Figure 5 A and 5B).

Sequenced reads from samples of autofeeding *C. reflexa* growing on tomato samples were quality-trimmed and Illumina adapter sequences were removed using Trimmomatic [37] with default settings. PolyA-tails were removed from the ends of reads and read pairs with 1 or both reads <75 bp were discarded. The surviving reads were subjected to a filtering pipeline using bwa 0.75 [38] (bwa aln –n 1 and otherwise default settings, followed by bwa sampe with default settings). In order to remove potential contamination by the tomato host plant, reads were aligned against ITAG2.3 cDNA [39]. The reads that survived this filter (i.e., neither read in a pair aligned properly to a filter sequence) were aligned against a database of common contaminants consisting of cDNA from *H. sapiens* (GRCh37.75) as well as fungal and *E. coli* sequences obtained from Refseq (July 25, 2014).

### Assembly of contigs using reads from not-in-contact tissues

For assembly of transcriptome sets of the nc tissues of *C. japonica* (Cj_nc_reads) and *I. balsamina* (Ib_nc_reads), these read sets were used as input for the Velvet software (version 1.2.10) [18] and, subsequently, Oases (version 0.2.08) [19]. The k-mer hash length of Velvet was set to 59, and –ins length of Oases was set to 175. The resulting transcript sets are referred to as Cj_nc_contigs and Ib_nc_contigs, respectively.

### Classification of the reads

*C. japonica* and *I. balsamina* reads among the CjIb_if_reads were classified using three sequential similarity searches (Figure 1). First, we performed *de novo* assembly of reads obtained from the nc samples as described above. CjIb_if_reads were mapped against the resulting transcript sets (Cj_nc_contigs and Ib_nc_contigs), using BLASTN (match length ≥ 90 bp, *e* value <1e-20, allowing for ≤1 mismatch and 1 gap). Reads that uniquely mapped to either Cj_nc_contigs or Ib_nc_contigs were regarded as reads originating from *C. japonica* (Cj_Ibif_reads) or *I. balsamina* (Ib_Cjif_reads), respectively. Reads that could not be mapped during this first step were mapped against unigene sets of the same genus (corresponding to *C. reflexa*, *C. pentagona* [17], and *C. suaveolens* [18]) using BLASTN (match length ≥90 bp, *e* value <1e-20, identity ≥ 90%). Reads matching any of these transcripts were classified as Cj_Ibif_reads. Reads that could not be mapped during this second step were used in a BLASTN search against the nt database (May 1, 2013). If the top five hits of a read were the entries from the Convolvulaceae plant species, then the read was classified as Cj_Ibif_reads. Analogously, if the top five hits were Balsaminaceae entries, then the read was classified as Ib_Cjif_reads. For *de novo* transcriptome assembly of *C. japonica* and *I. balsamina*, we selected all read pairs where classification of the mates was identical.

*C. japonica* and *G. max* reads among the CjGm_if_reads were classified using two approaches (Figure 5A and B). In the first approach, CjGm_if_reads were mapped against the contig sets — Cj_contigs_ci1 and Gmax_275_Wm82.a2.v1.transcript.fa.gz downloaded from the PhytozomeV10 (http://phytozome.jgi.doe.gov/pz/portal.html; match length ≥67 bp, *e* value <1e-20, allowing for ≤1 mismatch and 1 gap) [26]. Reads that uniquely mapped to Gmax_275_Wm82.a2.v1.transcript were regarded as reads originating from *G. max* (Gm_Cjif_reads1). Reads that uniquely mapped to Cj_contigs_ci1 were regarded as reads originating from *C. japonica* (Cj_Gmif_reads1). Reads that could not be mapped during this first step were mapped against unigenes of *Cuscuta* genus (match length ≥67 bp, *e* value <1e-20, identity ≥ 90%). Reads mapped to *Cuscuta*-genus unigenes were regarded as those originating from *C. japonica* and added to Cj_Gmif_reads1. Reads that could not be mapped during this second step were used in a BLASTN search against the nt database (May 1, 2013). If the top 5 hits of a read were the entries from the Convolvulaceae plant species, then the read was classified as Cj_Gmif_reads1.

In the second approach, reads derived from *G. max* in CjGm_if_reads were separated by mapping CjGm_if_reads onto Gmax_275_Wm82.a2.v1.transcript. Mapped reads were regarded as those derived from *G. max* (Gm_Cjif_reads2). Unmapped reads were regarded as those derived from *C. japonica*. The resulting Cj_Gmif_reads2 was used for assembly to obtain Cj_contigs_cg2.

### *De novo* transcriptome assembly

Assembly of *C. japonica* contigs using a merged dataset of Cj_nc_reads and Cj_Ibif_reads, and *I. balsamina* contigs using a merged dataset of Ib_nc_reads and Ib_Cjif_reads, were performed by using the Velvet software (version 1.2.10) [19] and, subsequently, Oases (version 0.2.08) [20]. The k-mer hash length of Velvet was set to 59, and –ins length of Oases was set to 175. The resulting transcript sets are referred to as Cj_contigs_ci1 or Ib_contigs, respectively. The read sets used for *de novo* assembly are available [DDBJ DRA: DRZ003178 and DRZ003179]. Sequence files of Cj_contigs_ci1 and Ib_contigs are available as Additional file 6 and Additional file 7.

In the *de novo* transcriptome assembly using Cj_Gmif_reads1 and Cj_Gmif_reads2, the same assembly procedure using Velvet/Oases as described above was used. The resulting transcript sets are referred to as Cj_contigs_cg1 and Cj_contigs_cg2, respectively. Sequence file of Cj_contigs_cg1 is available as Additional file 8.

In the *de novo* transcriptome assembly of *C. reflexa*, all read pairs from *C. reflexa* that survived the filtering pipeline described above were used for *de novo* transcriptome assembly using Trinity (version r20140717 with default parameters and –jaccard_clip option) [21].

### Functional annotation

Cj_contigs_ci1, Cj_contigs_cg1 and Cj_contigs_cg2 were searched against the plant protein database of refseqplant (*e* value <1e-5) using BLASTX [40]. GO annotations [41] were obtained from TAIR10 according to the similarity to *Arabidopsis thaliana* genes (ftp://ftp.arabidopsis.org/home/tair/Genes/TAIR10_genome_release/). The contig sets were further matched against unigenes of *C. pentagona* [17], *C. suaveolens* [18], *C. reflexa* (SRA: SRP038020), *Tryphysaria versicolor* (TrVeBC1 and TrVeBC2), *Striga hermonthica* (StHeBC1 and StHeBC2), and *Orobanche aegyptiaca* (OrAeBC4) (Parasitic Plant Genome Project http://ppgp.huck.psu.edu/) using BLASTN with *e* value <1e-5. Ib_contigs were used for BLASTX search against the plant protein database of refseqplant (*e* value <1e-5). According to the similarity to *Arabidopsis* genes, a GO annotation was obtained as described in Mochizuki et al. [40] using the GO dataset available at http://www.plant.osakafu-u.ac.jp/~ogata/downloadgo.html. Prediction of ORFs was performed with the OrfPredictor software [42]. Full-length transcripts were identified by testing whether both start and stop codon were detected within a contig’s sequence. The assembled *C. reflexa* contigs were checked for ORFs using an in-house Python script (https://github.com/cschu/fortuna). Contigs with an ORF of at least 200 bp were then searched against the plant protein database of refseqplant using BLASTX. GO annotation was then attempted for all contigs that matched against refseqplant using the same data sets described above. *C. reflexa* BLASTX runs were performed with the following parameters: *e* value <1e-5, ≥75% query coverage, >40% identity (identities + positives).

### Gene expression analysis and identification of differentially expressed genes

Cj_nc_reads and Cj_Ibif_reads were mapped to Cj_contigs_ci1, and Ib_nc_reads and Ib_Cjif_reads were mapped to Ib_contigs using BLASTN (parameter settings: match length ≥90 bp; ≤1 mismatch and 1 gap insertion allowed). Reads per kilobase per million mapped reads (RPKM) were calculated separately. Library size normalization and differential gene expression analysis were performed using the DESeq [43] and R software [44]. Cj_Gmif_reads1 and Cj_Gmif_reads2 were mapped to Cj_contigs_cg1 and Cj_contigs_cg2, respectively, using BLASTN (parameter settings: match length ≥90 bp; ≤1 mismatch and 1 gap insertion allowed). Gm_Cjif_reads1 and Gm_Cjif_reads2 were mapped to Gmax_275_Wm82.a2.v1.transcript using BLASTN (parameter settings: same as above). RPKM values were calculated separately.

### Assessment of read classification quality

To evaluate the degree of misclassification of reads with respect to their source organism, Cj_nc_reads and Ib_nc_reads were mapped to a merged transcript set consisting of Cj_contigs_ci1 and Ib_contigs using BLASTN (match length ≥90 bp; *e* value <1e-20, 1 mismatch and 1 gap insertion allowed). Cj_nc_reads that were mapped to Ib_contigs, and Ib_nc_reads that were mapped to Cj_contigs_ci1 were regarded as misclassified. To evaluate the rate of assignment of *C. japonica* reads to *I. balsamina*, or vice versa, Cj_nc_reads and Ib_nc_reads, respectively, were mapped to a merged transcript set consisting of Cj_nc_contigs and Ib_nc_contigs using BLASTN as described above. Cj_nc_reads that were mapped to Ib_nc_contigs as well as Ib_nc_reads mapped to Cj_nc_contigs were regarded as misclassified. For the binary classification of *C. japonica* reads and *I. balsamina* reads, the following 4 outcomes are possible. We defined a *C. japonica* read as a true positive (TP) if it was mapped to a *C. japonica* contig, and as a false negative (FN) if it was mapped to an *I. balsamina* contig. An *I. balsamina* read that was mapped to a *C. japonica* contig was defined as a false positive (FP). Finally, an *I. balsamina* read that was mapped to an *I. balsamina* contig was defined as a true negative (TN). For the nomenclature of *I. balsamina* reads, switch the term “*C. japonica*” and “*I. balsamina*” in the definition above. The true positive rate (TPR) was defined as TP / (TP + FN) and false positive rate (FPR) as FP / (TN + FP). The ROC AUC was calculated using the R package ROCR [45]. The same procedure was applied to evaluate the degree of misclassification between *C. japonica* and *G. max*.

### Cluster analysis

Read counts were normalized and subjected to identification of differentially expressed genes by using TCC and R software with FDR < 0.05 [46]. Clustering analysis of differentially expressed genes was performed by using function hclust from the R stats package [47].

### Light microscopy

The interface tissues were fixed with formalin, acetic acid:ethanol:water (90:5:5, v/v/v). Fixed samples were sliced into 80 – 100 micrometer-thick sections with the Vibratome (VIB-1500, Vibratome Co. Ltd., St. Louis, MO, USA). Histochemical staining of sections was performed using a 0.5% (w/v) solution of Toluindine Blue O (1B-481, Waldeck GmbH & Co., Munster, Germany) in distilled water. Stained slices were observed and photographs were taken by using the Biological Microscope BX51 (Olympus, Tokyo, Japan) with the CCD camera, VB-7010 (KEYENCE, Osaka, Japan).

## Additional files

Additional file 1:
**Total RNA yield from equal amount of fresh tissues of** ***C. japonica*** **and** ***I. balsamina*** **.**
Additional file 2:
**GO profiles of Cj_contigs_ci1 and Ib_contigs.** (A) Molecular Function. (B) Cellular Component. (C) Biological Process. Black bars: Cj_contigs_ci1. Grey bars: Ib_contigs. White bars: all *Arabidopsis* genes. Additional file 3:
**Similarity to the transcripts of other plants.** (A) *C. japonica* (Cj_contigs_ci1). (B) *I. balsamina* (Ib_contigs). Additional file 4:
**Differential expression of** ***C. japonica*** **contigs (Cj_contigs_ci1) and** ***I. balsamina*** **contigs (Ib_contigs) in the interface-region tissue compared to the not-in-contact tissue.**
Additional file 5:
**Differentially expressed genes of** ***C. japonica*** **(Cj_contigs_cg1) and** ***G. max*** **in the interface region.**
Additional file 6:
**A multi FASTA file of the Cj_contigs_ci1 (140519_Cj_ contigs_ci1.fa).**
Additional file 7:
**A multi FASTA file of the Ib_contigs (140519_Ib_contigs.fa).**
Additional file 8:
**A multi FASTA file of the Cj_contigs_cg1.**

## Abbreviations

Cj: *Cuscuta japonica*
Cr: *Cuscuta reflexa*
Ib: *Impatiens balsamina*
Gm: *Glycine max*
nc: not-in-contact
if: interface region
RPKM: Reads per kilobase per million mapped reads
ROC AUC: Receiver operating characteristic and its area under the curve
