# Supplementary material for: A bioinformatics approach to distinguish plant parasite and host transcriptomes in interface tissue by classifying RNA-Seq reads
Source: Plant Methods. 2015 May 3;11:34. doi: 10.1186/s13007-015-0066-6 (PMC4458054; doi:10.1186/s13007-015-0066-6)
Supplement: Additional file 1: — Total RNA yield from equal amount of fresh tissues of C. japonica and I. balsamina . [file 13007_2015_66_MOESM1_ESM.pdf]

Additional file 1. Total RNA yield from equal amount of fresh tissues of *C. japonica* and *I. balsamina.*

Cuscuta japonica		
Fresh weight (mg)	Total RNA (μg)	Total RNA per mg fresh weight (μg $mgFW^{-1}$)
102	29.7	0.29
63.7	13.4	0.21

Impatiens balsamina		
Fresh weight (mg)	Total RNA (μg)	Total RNA per mg fresh weight (μg $mgFW^{-1}$)
114	1.70	0.015
115	1.38	0.012
109	2.08	0.019
111	2.52	0.022
110	1.01	0.0092
215	4.78	0.022
