# Supplementary figures and images for: A bioinformatics approach to distinguish plant parasite and host transcriptomes in interface tissue by classifying RNA-Seq reads
Source: Plant Methods. 2015 May 3;11:34. doi: 10.1186/s13007-015-0066-6 (PMC4458054; doi:10.1186/s13007-015-0066-6)

A

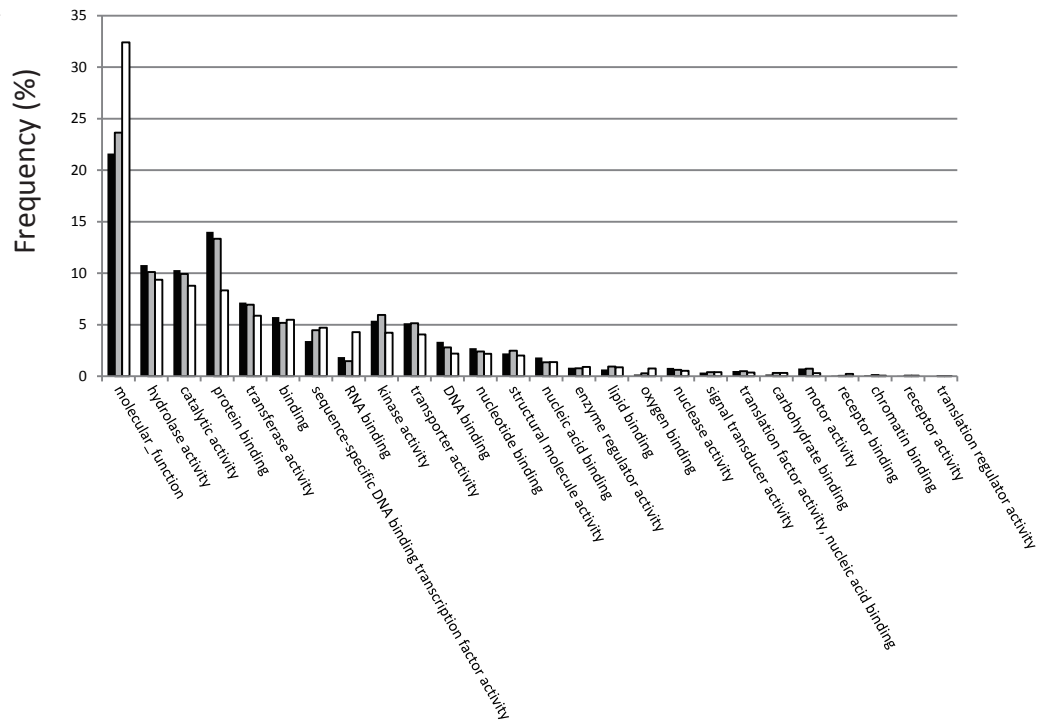

B

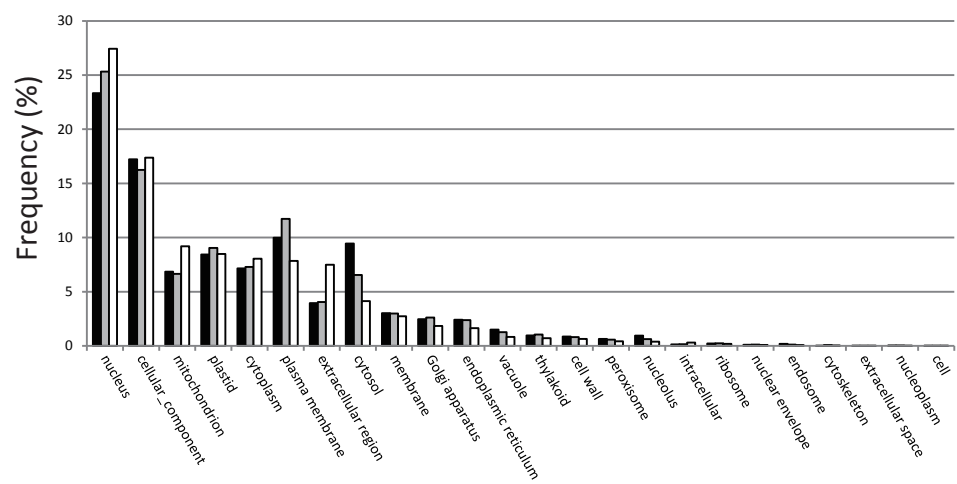

C

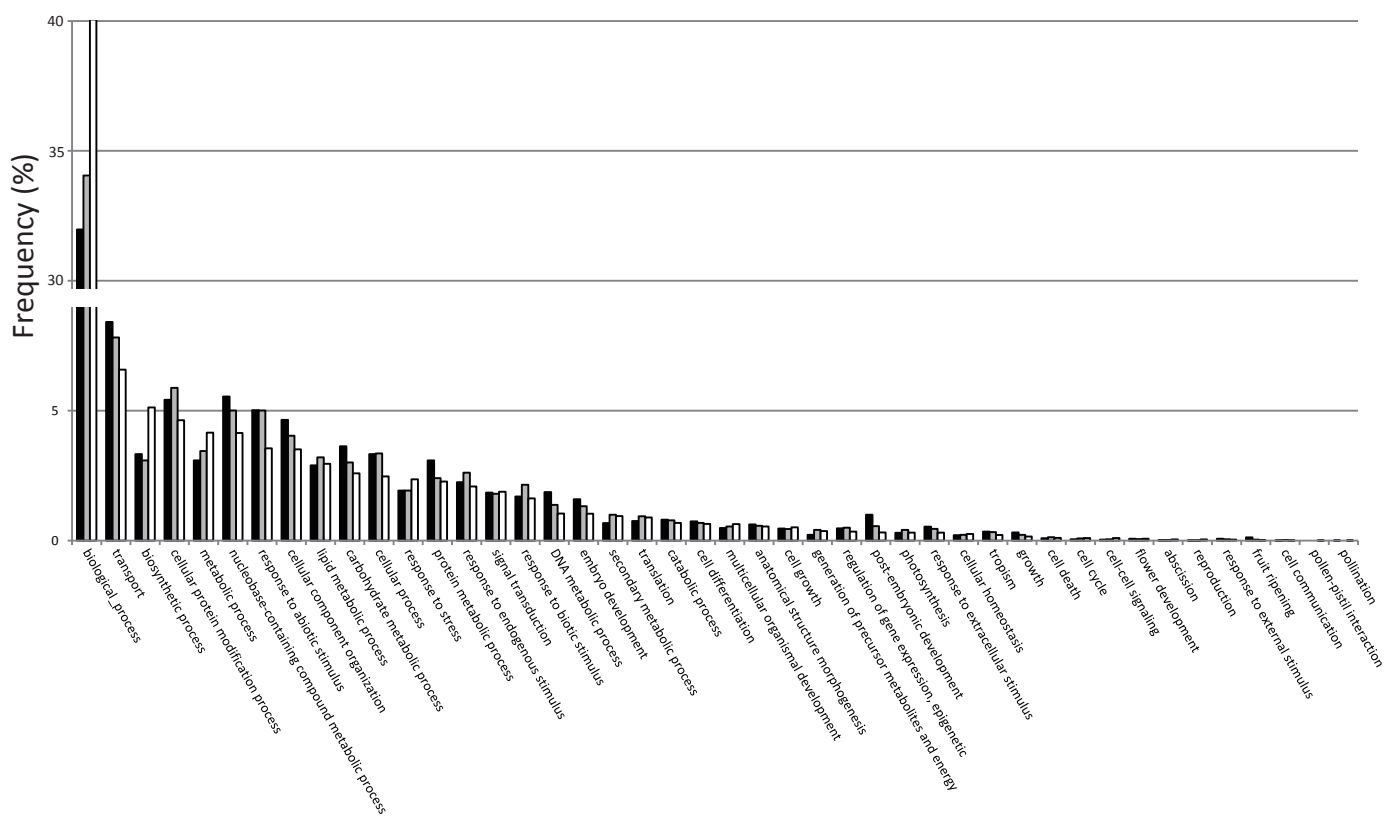

Supplement: Additional file 2: — GO profiles of Cj_contigs_ci1 and Ib_contigs. (A) Molecular Function. (B) Cellular Component. (C) Biological Process. Black bars: Cj_contigs_ci1. Grey bars: Ib_contigs. White bars: all Arabidopsis genes. [file 13007_2015_66_MOESM2_ESM.pdf]

**A**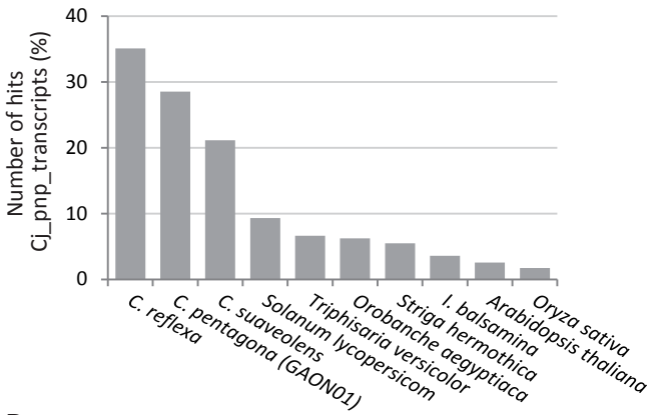**B**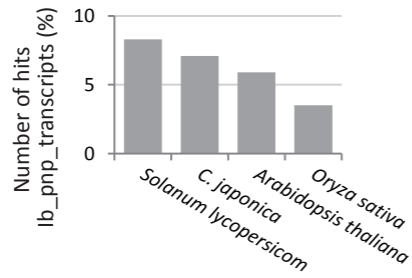

Supplement: Additional file 3: — Similarity to the transcripts of other plants. (A) C. japonica (Cj_contigs_ci1). (B) I. balsamina (Ib_contigs). [file 13007_2015_66_MOESM3_ESM.pdf]
